# Facet Dependence of Biosynthesis of Vivianite from Iron Oxides by *Geobacter sulfurreducens*

**DOI:** 10.3390/ijerph20054247

**Published:** 2023-02-27

**Authors:** Xiaoshan Luo, Liumei Wen, Lihua Zhou, Yong Yuan

**Affiliations:** 1 Guangdong Key Laboratory of Environmental Catalysis and Health Risk Control, School of Environmental Science and Engineering, Institute of Environmental Health and Pollution Control, Guangdong University of Technology, Guangzhou 510006, China; 2 Department of Pharmaceutical Engineering, School of Biomedical and Pharmaceutical Sciences, Guangdong University of Technology, Guangzhou 510006, China

**Keywords:** iron oxide, exposed facet, dissimilated iron reduction, vivianite

## Abstract

Vivianite plays an important role in alleviating the phosphorus crisis and phosphorus pollution. The dissimilatory iron reduction has been found to trigger the biosynthesis of vivianite in soil environments, but the mechanism behind this remains largely unexplored. Herein, by regulating the crystal surfaces of iron oxides, we explored the influence of different crystal surface structures on the synthesis of vivianite driven by microbial dissimilatory iron reduction. The results showed that different crystal faces significantly affect the reduction and dissolution of iron oxides by microorganisms and the subsequent formation of vivianite. In general, goethite is more easily reduced by *Geobacter sulfurreducens* than hematite. Compared with Hem_{100} and Goe_L{110}, Hem_{001} and Goe_H{110} have higher initial reduction rates (approximately 2.25 and 1.5 times, respectively) and final Fe(II) content (approximately 1.56 and 1.20 times, respectively). In addition, in the presence of sufficient PO_4_^3−^, Fe(II) combined to produce phosphorus crystal products. The final phosphorus recoveries of Hem_{001} and Goe_H{110} systems were about 5.2 and 13.6%, which were 1.3 and 1.6 times of those of Hem_{100} and Goe_L{110}, respectively. Material characterization analyses indicated that these phosphorous crystal products are vivianite and that different iron oxide crystal surfaces significantly affected the size of the vivianite crystals. This study demonstrates that different crystal faces can affect the biological reduction dissolution of iron oxides and the secondary biological mineralization process driven by dissimilatory iron reduction.

## 1. Introduction

With the growing world population, the demand for phosphorus is also increasing, and the problems of phosphorus pollution and the phosphorus crisis are becoming increasingly prominent [1]. The potential of vivianite (Fe_3_(PO_4_)_2_·8H_2_O), a type of ferrous phosphate mineral, to alleviate both issues has recently attracted wide attention [2]. Many studies have shown that phosphate can be removed or recovered by vivianite crystallization in anaerobic wastewater or anaerobic sludge digesters. In particular, the formation of vivianite through biomineralization induced by dissimilatory iron-reducing bacteria (DIRB) has attracted significant attention [3,4]. Under anaerobic conditions, Fe(III) minerals are reduced to Fe(II) through the actions of DIRB. When Fe(II) and co-existing PO_4_^3−^ in the system reach the corresponding solubility product (Ksp), vivianite will be generated [5,6,7]. Vivianite is not only an effective way to recover phosphorus from wastewater but also has considerable economic value. It can be used as a raw material for lithium iron phosphate (LiFePO_4_) battery electrodes or pigments [4]. Vivianite has also attracted significant attention due to its properties in soil environments, such as soil heavy metal solidification, dechlorination of organic matter, phosphorus fixation in sediment, and as an agricultural slow-release fertilizer.

At present, the main limitations in the bioreductive synthesis of vivianite for phosphorus recovery are the low reduction efficiency of Fe(III) in the reaction system and the small crystal size of the produced vivianite. The process of vivianite bioformation is often affected by many complex factors, including microorganisms, pH, and the Fe/P molar ratio. Previous research has shown that the pH value of the reaction system plays an essential role in the formation of vivianite because both phosphate and Fe(II) depend on this factor. Liu et al. pointed out that compared with pH values of 6.0 and 9.0, the crystallization of vivianite is more favorable at pH 7.0 [8]. In addition, Wang et al. studied the effects of different Fe/P ratios (1, 1.5, 3) on the formation efficiency of vivianite. Their results showed that an Fe/P molar ratio of 1 is more conducive to the reduction of iron and the formation of vivianite because the appropriate concentration of phosphate can provide enough nutrients for microbial growth in addition to substrate for mineralization [9]. Other studies have shown that adding conductive materials, such as graphite [2] and quartz sand [10], to the reaction system can promote the electron transfer between iron oxides and DIRB, thus improving the reduction rate of iron and expanding the crystal size of blue iron. However, it is not clear how the different crystal faces of ferric oxide affect the microbial-dissimilated iron reduction and thus drive the synthesis of vivianite.

Therefore, several forms of crystalline hematite, (Hem_{100}, Hem_{001}), goethite (Goe_H{110}, and Goe_L{110}), were prepared and characterized using XRD, SEM, and BET in this study. An anaerobic iron–phosphorus complex system was constructed with different crystal surface iron oxides as electron acceptors and potassium dihydrogen phosphate as the phosphorus source to explore the differences in bioavailability and product formation efficiency from the different crystal faces of iron oxide minerals in a *Geobacter sulfurreducens* system and the effect of the electron shuttle AQDS on dissimilatory iron reduction. The reduction rate of iron and the phosphorus recovery rate were analyzed, and the final phosphorus crystallization product was characterized using XRD, SEM, and EDS. The effects of different crystal faces on the synthesis of vivianite driven by dissimilated iron reduction were investigated.

## 2. Materials and Methods

### 2.1. Synthesis of Iron Oxide Nanocrystals

Two hematite materials with preponderantly exposed {001} and {100} faces (denoted as Hem_{001} and Hem_{100}) and two goethite materials with different contents of exposed {110} facet (denoted as Goe_H{110} and Goe_L{110}) were synthesized, respectively. Details of the synthesized nanoparticles are provided in the Supporting Information (Text S1) [11,12].

### 2.2. Geobacter Sulfurreducens PCA Inoculation

*Geobacter sulfurreducens* (*G. sulfurreducens*, PCA) was used as the model microorganism in this study. *G. sulfurreducens* was grown in 50 mL serum bottles at 30 °C in an anoxic NBAF medium containing CaCl_2_·2H_2_O (0.04 g L^−1^), MgSO_4_·7H_2_O (0.10 g L^−1^), NaHCO_3_ (1.80 g L^−1^), Na_2_CO_3_·H_2_O (0.50 g L^−1^), Na_2_SeO_4_ solution (1 mL L^−1^), NB salts (10 mL L^−1^), vitamin solution (1 mL L^−1^), and mineral solution (10 mL L^−1^) [13]. Sodium acetate (15 mM) was used as the electron donor, while fumaric acid (40 mmol L^−1^) was served as the electron acceptor. All reagents in the experiment were of pure analytical grade. The medium was adjusted to the final pH of 6.9–7.0 and flushed with N_2_/CO_2_ (80:20, *v*/*v*) gas mixture. The serum bottles were sealed with butyl rubber and an aluminum cover and sterilized at 121 °C for 30 min. Approximately seven days after inoculation, *G. sulfurreducens* cells were obtained during the logarithmic growth phase (OD600 ≈ 0.3) by centrifugation at 4000 rpm (15 min, 4 °C) for follow-up experiments.

### 2.3. Batch Trials for Microbial Fe(III) Reduction and Biosynthetic Vivianite

For the microbial Fe(III) reduction experiments, *G. sulfurreducens* cells were harvested and washed twice with bicarbonate buffer. Aliquots of the washed cells (~10^8^ cells mL^−1^) were injected into 50 mL serum bottles with 25 mL anoxic sterile NBAF medium. Sodium acetate (15 mM) was used as the electron donor, while iron oxide (1 g L^−1^) served as the electron acceptor. Next, 9,10-anthraquinone-2,6-disulfonic acid disodium salt (AQDS; 50 μM) was added to the serum bottles to act as an electron shuttle. Bottles were incubated (stationary) at 30 °C in the dark for 30 days.

For the vivianite biosynthetic experiments, the batch systems were conducted in 50 mL serum bottles with 25 mL medium containing sodium acetate (15 mM), iron oxides (9 mM as Fe atoms), KH_2_PO_4_ (9 mM), KCl (0.13 g L^−1^), NH_4_Cl (0.31 g L^−1^), vitamin solution (5 mL L^−1^), and mineral solution (12.5 mL L^−1^). The medium was adjusted to pH 7.6 and flushed with N_2_ for 30 min to ensure an anaerobic environment. *G. sulfurreducens* cells were harvested and washed twice using phosphate buffer, and aliquots of the washed cells (~10^8^ cells mL^−1^) were injected into the prepared anoxic sterile medium. Bottles were placed inside a shaking incubator at 200 rpm and cultivated at 30 °C in the dark for 30 days. All tests were performed in triplicate.

### 2.4. Chemical Analysis

Samples were periodically taken from each bottle with sterile syringes. The soluble Fe^2+^ and Fe^3+^ concentrations were determined using 1,10-phenanthroline spectrophotometry [14], and soluble PO_4_^3−^ was measured according to a modified molybdenum antimony anti-spectrophotometry method, as described by Uhlmann et al. [15] The reacted solid samples were pretreated in 3M HCl overnight, and then P, Fe(II), and Fe(III) content were measured using the methods described above.

The concentration of total Fe(III) (*C*_TFe(III)_) and total Fe(II) (*C*_TFe(II)_) and the Fe(III) reduction rate (R_Fe_) were calculated as follows:

(1) $$C_{\mathrm{TFe}\left(\mathrm{III}\right)}=C_{\mathrm{Fe}\left(\mathrm{III}\right)}+C_{{\mathrm{Fe}}^{3+}}$$

(2) $$C_{\mathrm{TFe}\left(\mathrm{II}\right)}=C_{\mathrm{Fe}\left(\mathrm{II}\right)}+C_{{\mathrm{Fe}}^{2+}}$$

(3) $$R_{\mathrm{Fe}}=\frac{d(C_{\mathrm{TFe}\left(\mathrm{II}\right)})}{dt}$$

where *C*_Fe(III)_ and *C*_Fe(II)_ are the solid Fe(III) and Fe(II) contents, respectively, and *C*_Fe_^2+^ and *C*_Fe_^3+^ are the concentrations of Fe^2+^ and Fe^3+^, respectively, and *t* is the inoculation time (day) [9].

The calculation of phosphorus removal efficiency (RP) and vivianite formation efficiency (RV) are detailed in the Supporting Information (Text S2).

### 2.5. Characterization Analysis

Brunauer–Emmett–Teller specific surface area (SABET) of synthetic iron oxide was tested using Micromeritics ASAP 2460 surface area analyzer, the calculation formula is explained in detail in Supporting Information Text S2. The crystal structures of iron oxide nanoparticles and biogenic secondary minerals were determined using an X-ray diffractometer (XRD, Bruker D8, Tokyo, Japan) under Cu-Kα radiation at an accelerating voltage of 40 kV and a filament current of 40 mA. MDI Jade software (version 6.0) was used to identify the minerals. Scanning electron microscopy (SEM, SU8010, HITACHI, Tokyo, Japan) was applied to observe the morphology and elemental composition of the samples. The protein content was extracted using a bacterial active protein extraction kit (C500023-0020, Sangon Biotech Co., Shanghai, China) and quantified using a modified Bradford protein assay kit (C503041-1000, Sangon Biotech Co., China). Each sample was assayed in triplicate, and the results presented with the mean values.

## 3. Results and Discussion

### 3.1. Characteristics of Iron Oxides with Different Facet Structures

**Figure 1:**
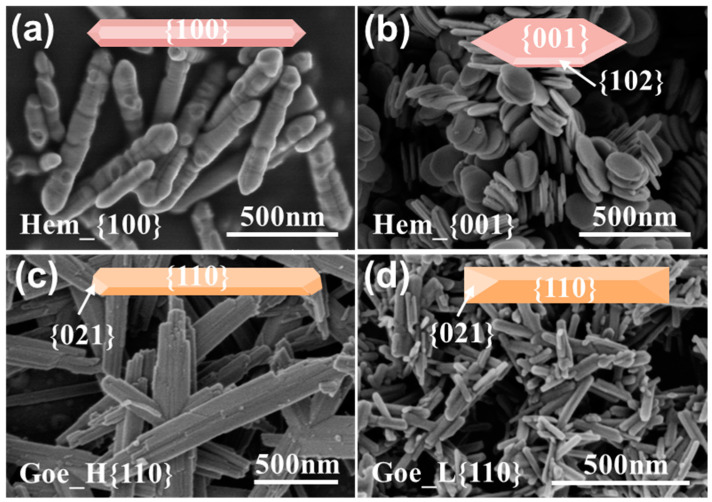
SEM images of synthetic iron oxide. (**a**) Hem_{100}, (**b**) Hem_{001}, (**c**) Goe_H{110}, and (**d**) Goe_L{110}.

XRD analysis was applied to confirm the iron oxide nanomaterial crystallographic structure and phase purity. As shown in Figure S1a–c, the results confirmed that the synthetic powders could be assigned to hematite α-Fe_2_O_3_ (JCPDS No. 33-0664) and goethite α-FeOOH (JCPDS No. 29-0713), respectively, with pure crystalline phases and high crystallinity [11,16,17]. The crystallinity of Hem_{100}, Hem_{001}, Goe_H{110}, and Goe_L{110} were calculated to be 52.69, 51.30, 42.98, and 48.48%, respectively, indicating that hematite had a slightly higher crystallinity than goethite. The SEM images showed that Hem_{100} was hexagonal prisms with an average length of 750 nm and a mean width of 120 nm, while Hem_{001} was hexagonal plates with a mean length of 180 nm and an average thickness of 20 nm (Figure 1a,b and Table S1). The nanoparticles synthesized in the experiment were consistent with the regular morphology of the corresponding single crystal. Moreover, the morphology of Goe_H{110} was bamboo-strip-like nanoneedles with an average length of 1300 nm and a mean width of 215 nm, while that of Goe_L{110} was nanorods with an average length of 120 nm and a mean width of 35 nm (Figure 1c,d and Table S1). Moreover, according to previous studies [11,16], the dominant exposed surface of Hem_{100} were {100} facets (about 94.0%), whereas that of Hem_{001} were {001} facets (about 85.5%). The exposed facets of Goe_H{110} were composed of approximately 98.5 {110} and 1.5% {021}, while that of Goe_L{110} were composed of approximately 82.3 {110} and 17.7% {021}. The BET surface area of Hem_{100}, Hem_{001}, Goe_H{110}, and Goe_L{110} were 8.04, 24.59, 66.88, and 76.01 m^2^ g, respectively, as determined using a N_2_ adsorption-desorption measurement (Table S1).

### 3.2. Facet Dependence of Bioreduction of Iron Oxides by Geobacter sulfurreducens

**Figure 2:**
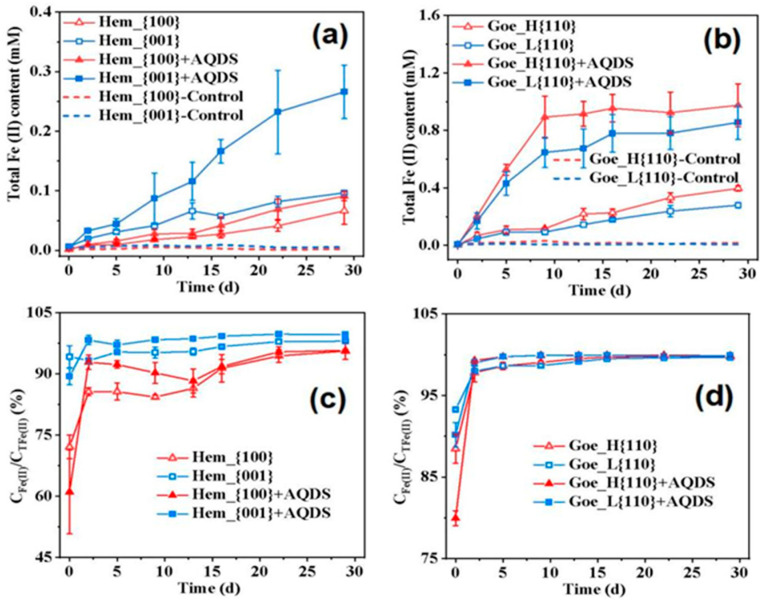
Concentration of the reduced Fe(II) produced from microbial Fe(III) reduction (**a**,**b**). Percentage of solid Fe(II) content in the total reduced Fe(II) content (**c**,**d**). Data correspond to an average of biological triplicates, and the error bars indicate standard deviations. Experimental conditions: bicarbonate−buffered (20 mM); *Geobacter sulfurreducens* (OD_600_ ≈ 0.30); 0 or 50 μm AQDS; 0.10 g/L iron oxide nanocrystals.

In order to evaluate the effect of the exposed surface of iron oxides on the Fe(III) reduction process of *G. sulfurreducens*, different crystal surface iron oxides were used as electron acceptors and sodium acetate as electron donors. The differences in the *G. sulfurreducens* reduction of iron oxides in different crystal surfaces were evaluated using the determination of aqueous Fe(II) and solid Fe(II) with and without electron shuttle (AQDS). Fe(II) production during dissimilatory iron reduction by *G. sulfurreducens* of iron oxides with different exposed facets was examined in the absence/presence of AQDS. As shown in Figure 2a,b, no apparent Fe(II) was detected in uninoculated serum bottles (abiotic controls). In contrast, apparent Fe(II) formation was observed in all inoculated serum bottles, suggesting the iron reduction process was mediated by *G. sulfurreducens*. The extent of reduction for Hem_{100} and Hem_{001} increased with culture time (Figure 2a). In the absence of AQDS, the Fe(III) initial reduction rate was about 0.01 mmol L^−1^ d^−1^ for Hem_{001}, which was approximately 2.25 times higher than Hem_{100}. After 29 days, the total Fe(II) contents were about 0.07 mmol L^−1^ and 0.10 mmol L^−1^ for Hem_{100} and Hem_{001}, respectively. The extent of reduction for Goe_H{110} and Goe_L{110} is shown in Figure 2b. In the absence of AQDS, the initial reduction rate for Goe_H{110} was about 0.03 mmol L^−1^ d^−1^, which was approximately 1.50 times higher than that for Goe_L{110}, and the total Fe(II) contents at 29 days were about 0.40 mmol L^−1^ and 0.28 mmol L^−1^ for Goe_H{110} and Goe_L{110}, respectively. A similar result was found to those of hematite. All of these results suggest that the Hem_{001} surface was more easily reduced by *G. sulfurreducens* than the Hem_{100} surface without AQDS. This was similar to the results reported by predecessors. Hu et al. [18] showed that because the Hem_{001} surface had a stronger oxidizing surface and superior electrical conductivity than the Hem_{100} surface, it showed a stronger affinity with Shewanella putrefaciens CN-32 and was easier to reduce. Goe_H{110} also showed a higher *G. sulfurreducens* reduction rate and degree than Goe_L{110}. In addition, as goethite showed lower crystallinity and relatively larger solubility, it was easier to be utilized by microorganisms. Therefore, the microbial reduction degree of goethite was greater than that of hematite [19,20]. The active site of surface reactivity played a key role in the bioavailability and reactivity of iron oxide. Previous studies showed that the initial rate and long-term degree of microbial iron (III) oxide reduction was controlled by the specific surface area and reaction site density of the solid phase [21]. Therefore, the larger the specific surface area of iron oxide, the more surface active sites there are, and the more conducive iron oxides are to microbial reduction and dissolution [22]. However, the morphology of hematite is hexagonal plates, while the morphology of goethite is bamboo needles. The surface area of goethite is larger than that of hematite with the same weight; this is another reason why the microbial reduction of goethite is greater than that of hematite.

Fe(II) production from both forms of goethite increased in the presence of AQDS. However, the extent of goethite reduction gradually stabilized after nine days, possibly due to the large amount of Fe(II) formation limiting the extent of iron(III) oxide reduction. Previous studies have indicated that the Fe(II) adsorbed on the surface of iron(III) oxides and dissimilatory Fe(III)-reducing bacteria (FeRB) could restrict the initial rate and long-term extent of microbial iron reduction by decreasing the number of surface reactive sites [21]. This result was further supported by the observations that Fe(III) reduction can be reactivated if absorbed Fe(II) is removed from the oxide surface or if new FeRB cells are added to the reactor [23]. All of these results suggest that AQDS, as an electron shuttle, could enhance the initial rate and degree of microbial iron trivalent reduction. However, when reduced Fe(II) reaches a certain concentration, the microbial reduction rate no longer increases, and the Fe(II) content becomes gradually stable [24].

Moreover, as shown in Figure 2c,d, based on these observations, we calculated the percentage of solid Fe(II) from the total Fe(II) content (*C*_Fe(II)_/*C*_TFe(II)_). The result showed that most of the Fe(II) (96–99%) was retained within the solid phase rather than in the aqueous phase at the end of the experiment. In conclusion, both in the presence or absence of AQDS, Hem_{001} was more easily reduced than Hem_{100} by *G. sulfurreducens*, which was in accordance with previous studies which demonstrated that the facet {001} is more readily bioavailable than facet {100} to *Shewanella oneidensis* [19,25]. Furthermore, Goe_H{110} led to higher initial rates and extents of Fe(III) microbial reduction than Goe_L{110}. Furthermore, compared with hematite, goethite was more likely to be reduced by *G. sulfurreducens*, which may have contributed to the higher solubility and lower crystallinity of goethite [20,21].

Changes in the crystal structure and morphology of the iron oxide following incubation with *G. sulfurreducens* in the presence and absence of AQDS after 30 days were characterized using XRD and SEM. As shown in Figure S2a,b, for both Hem_{100} and Hem_{001}, the diffraction peaks were consistent with the standard card of hematite, and almost no other new phases were observed. Previous studies have reported similar results in that the microbial Fe(III) reduction process of hematite did not cause any significant change in the crystal structure [24]. Compared with hematite, new phases were found in the XRD patterns of both Goe_H{110} and Goe_L{110} as a result of extended incubation, especially with ADQS treatment, with greater Fe(III) reduction, while the prominent diffraction peaks were consistent with the standard card of goethite (Figure S2c,d). Given that all reduction experiments were carried out in bicarbonate buffer, these phases may be composed of iron carbonate [26]. The SEM images of the Hem_{100} and Hem_{001} samples indicated they maintained their overall morphology, but slight defects could be observed on the surfaces of Hem_{001} (Figure S3a,b). This further indicates that both hematite samples retained their original crystal structures. However, the morphology of both goethite forms changed significantly after microbial reduction, especially with the addition of AQDS. Obvious fractures were observed in Goe_H{110}, while a significant size reduction was observed in Goe_L{110} (Figure S3c,d).

### 3.3. Facet-Dependent Formation of Vivianite during Dissimilatory Iron Reduction

**Figure 3:**
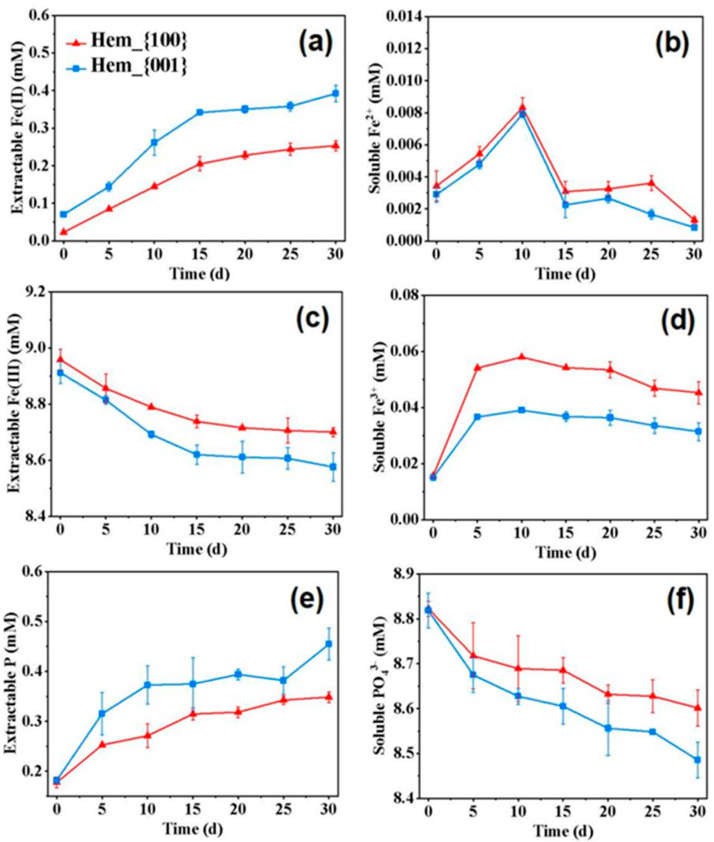
Variation of the (**a**) soluble Fe^2+^ content, (**b**) solid Fe(II) content, (**c**) soluble Fe^3+^ content, (**d**) solid Fe(III) content, (**e**) PO_4_^3+^ content, and (**f**) solid P content in hematite systems. Data correspond to an average of biological triplicates, and the error bars indicate standard deviations. Experimental conditions: hydrophosphate−buffered (9 mM); *Geobacter sulfurreducens* (OD_600_ ≈ 0.30); iron oxide nanocrystals (9 mM).

As shown in Figure 3 and Figure S4, with inoculations of *G. sulfurreducens*, the Fe(III) and PO_4_^3−^ content decreased in both the hematite and goethite systems, accompanied by the continuous increase of Fe(II) and P content in the solid phase. Specifically, during the initial five days, the R_Fe_ of the Hem_{001} group was about 0.03 mmol L^−1^ day^−1^ and 1.50 times that of the Hem_{100} group. Simultaneously, the R_Fe_ of the Goe_H{110} group was about 0.13 mmol L^−1^ day^−1^, which was 1.44 times that of the Goe_L{110} group (Figure S5). After 30 days, the Fe(II) content in the hematite gradually reached 0.25–0.39 mmol L^−1^ (Figure 3a). In contrast, the Fe(II) content in the goethite systems sharply increased during days 0–15 and approached 1.22–1.46 mmol L^−1^ (Figure S4a). These results indicated that Hem_{001} had a higher reduction rate and degree than Hem_{100} in the phosphoric acid buffering reaction system, so it may be more conducive to the formation of cyanite. As shown in Figure 3c, the Fe(III) content in the solid phase gradually decreased over time, indicating that hematite was constantly being dissolved and utilized or transformed in the reaction with microorganisms. The Fe(III) content in the solid phase greatly changed from 0 to 15 days, and the decrease of Fe(III) in the Hem_{001} system was more than that in Hem_{100}, which corresponded to the change of Fe(II) content in the solid phase.

In all systems, the concentration of reduced soluble Fe^2+^ remained around the range of 0–0.01 mmol L^−1^, indicating that Fe^2+^ did not accumulate in the system, which was mainly due to Fe^2+^ being quickly converted to other forms (Figure 3b and Figure S4b) [2]. In 0–10 days, the Fe^3+^ concentration increased rapidly and then decreased slightly due to the dissolution of iron oxide. It may be because the iron oxide minerals were partially dissolved under the action of microorganisms, in which Fe^3+^ was reduced to Fe^2+^. Fe^2+^ was further combined with PO_4_^3−^ to form Fe(II)-p precipitation (Figure 3d and Figure S4d) [2]. During the microbial dissimilatory iron reduction process in all systems, Fe(II) was released from the iron oxides and immediately reprecipitated with PO_4_^3−^ [27], resulting in a decrease in the concentration of PO_4_^3−^ (Figure 3f and Figure S4f) and an increase in the content of extractable P (Figure 3e and Figure S4e). After 30 days, the P content of Hem_{100} and Hem_{001} was 0.35 ± 0.01 mmol L^−1^ and 0.45 ± 0.03 mmol L^−1^, respectively (Figure 3e), while that of Goe_H{110} and Goe_L{110} was 1.18 ± 0.13 mmol L^−1^ and 0.76 ± 0.10 mmol L^−1^, respectively (Figure S4e). The continual and smooth increase in the solid phase Fe(II) and P indicated that vivianite was constantly accumulated. The final Rp of Hem_{100} and Hem_{001} was about 3.95 and 5.15%, respectively, while that of Goe_H{110} and Goe_L{110} was about 13.63 and 8.65%, respectively (Figure S5). Correspondingly, the Rv of Hem_{100}, Hem_{001}, Goe_H{110}, and Goe_L{110} was about 1.88, 2.90, 10.78, and 9.81%, respectively (Figure S5). The highest vivianite formation efficiency was obtained in the hematite system with predominantly exposed {001} facet and the goethite system with high contents of exposed {110} facet [28]. Compared with Hem_{100}, Hem_{001} was more easily dissolved by *G. sulfurreducens*, which could produce more Fe(II) to combine with PO_4_^3−^ and promote the formation of more phosphorus crystal products. In addition, studies showed that different crystal faces would affect the type and abundance of surface iron atoms and the number of surface active groups due to the different arrangement of atoms [29]. It was reported that the adsorption affinity of orthophosphate ions and iron oxides was closely related to the number of -FeOH groups on the surface [30], while previous studies showed that the density of -FeOH groups on the {001} surface of hematite was significantly higher than that on the {100} surface, which may be the reason for the higher phosphorus recovery of Hem_{001} [16]. In addition, the {110} surface of goethite was the dominant plane for phosphate adsorption [1], so the high proportion of the {110} surface of goethite may contain a higher density of -FeOH groups, which was more conducive to promoting the formation of phosphorus crystallization products.

**Figure 4:**
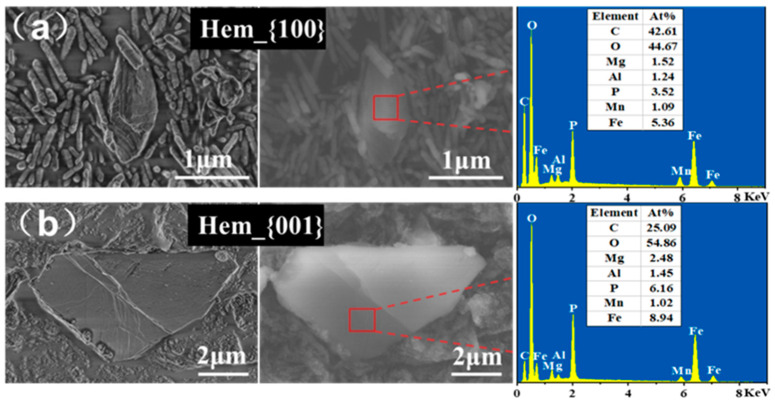
SEM image of vivianite in Hem_{100} (**a**) and Hem_{001} (**b**) systems and the corresponding EDS patterns.

**Figure 5:**
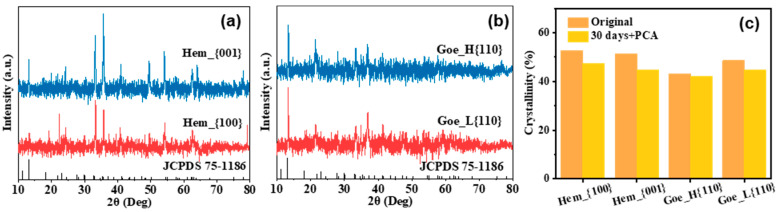
X-ray diffraction (XRD) patterns of phosphorus crystalline products collected 30 days after inoculation of (**a**) hematite and (**b**) goethite nanoparticles and (**c**) the crystallinity statistics. Experimental conditions: hydrophosphate-buffered (9 mM); *Geobacter sulfurreducens* (OD_600_ ≈ 0.30); iron oxide nanocrystals (9 mM).

The flower-like nodules composed of rhombic platy-shaped crystals stacked in random directions were vivianite (Figure 4 and Figure S6) and presented a morphology similar to that observed in a previous study [2]. Different exposed facets of iron oxide will affect the size of the vivianite crystal. Specifically, in the hematite experiments, biomineralized vivianite induced by dissimilatory Hem_{001} reduction had a larger particle size than Hem_{100} (Table S2). In the goethite experiments, vivianite in Goe_H{110} exhibited a larger aspect ratio than Goe_L{110} (Table S2). The EDS scans of these particles indicated that iron and phosphorus were the main distinguishing elements present, with Fe/P molar ratios comprised between 1.42 and 1.52 (1.5 in pure vivianite) (Table S2). Meanwhile, the XRD patterns (Figure 5) of collected precipitates revealed that the main diffraction peaks coincided with the vivianite reference pattern (JCPDS 75-1186), confirming that vivianite was present in all samples. SEM-EDS and XRD analyses suggested that vivianite was the primary detectable Fe-P mineral. Our results revealed that vivianite could be generated biogenetically by *G. sulfurreducens*, and its formation was strongly correlated to the exposed facets of iron oxide. In addition, the variation in the protein content indicated that different exposed facet types had little impact on microbial growth, with the biomass in Hem_{100}, Hem_{001}, Goe_H{110}, and Goe_L{110} groups increasing from 293.77 ± 9.38 mg L^−1^ to 1583.51 ± 13.78, 1577.23 ± 79.79, 1528.31 ± 4.85 mg, and 1519.83 ± 8.90 mg L^−1^ (Figure S7), respectively, indicating that different exposed facets of iron oxide had little impact on the biomass proliferation.

### 3.4. Potential Mechanism of Facet-Dependent Vivianite Formation by G. sulfurreducens

Iron oxide minerals have high reactivity and can be used as conversion centers for various inorganic and organic ions. The surface reactivity is usually determined by the ability of the surface hydroxyl (OH) group to interact/exchange with the solute/water. The surface OH group can be distinguished as a single (≡FeOH, -OH), double (≡Fe_2_OH, μ-OH), or triple (≡Fe_3_OH, μ_3_-OH) hydroxyl group based on the number of Fe atoms in the coordination lattice [28,31]. Among them, the -OH group is the main adsorption center of the ligand, the μ-OH group can exchange at higher loads, and the μ_3_-OH group is elastic to any ligand exchange reaction to a large extent. Previous experiments showed that the difference in the phosphate adsorption capacity of iron oxide minerals is mainly related to the mineral -OH site density. The affinity of the -OH group is 2–4 orders of magnitude stronger than that of the μ-OH group, which only becomes important under maximum load [32]. The Hem_{001} plane accounts for about 85.5% of the total surface area and contains the -OH, μ-OH, and μ_3_-OH groups, of which the -OH sites are more abundant [32]. The Hem_{100} plane accounts for about 94.0% of the total surface area and contains only the μ-OH group [32]. This can explain why the R_V_ of the Hem_{001} system was higher than that in the Hem_{100} system. Previous studies also showed that phosphates preferentially adsorbed on multidomain goethite particles exhibiting major {110} and {100} planes rather than single-domain goethite particles exhibiting more terminal{021} planes [33,34]. As stated above, the exposed surface {110} of Goe_H{110} accounted for about 98.5% and {021} accounted for 1.5%, while the exposed surface {110} of Goe_L{110} accounted for about 82.3% and {021} accounted for 17.7%. This explained why the R_V_ of Goe_H{110} was higher than that of Goe_L{110}. As DIRB, *G. sulfurreducens* uses iron (III) oxide as an electron acceptor for metabolism. The availability of Fe(III) to the cell limits electron transfer from DIRB to Fe(III) minerals [35]. The insolubility of iron oxide minerals further inhibits iron reduction in the periplasm or cytoplasm by limiting the movement of electrons across cell membranes [2]. The resulting lower reduction rate limits microbial bioavailability. Previous studies show that crystalline goethite and hematite solubility are less than 9 and 1.2%, respectively [36]. This is why the R_V_ in Geo_H{110} and Geo_L{110} systems were higher than the Hem_{001} and Hem_{100} systems. In addition, compared with Hem_{100}, Hem_{001} significantly improves the expression of cytochrome c and nanowire-like-related genes, which can significantly improve extracellular electron transport efficiency between iron oxide minerals and *G. sulfurreducens* to a great extent [37]. It is beneficial to improve the R_V_ in the Hem_{001} system. We plan to investigate the effects of goethite on the expression of cytochrome c and nanowires in the future. We speculated that different crystalline goethite (Geo_H{110} and Geo_L{110}) would also be similar to hematite (Hem_{001} and Hem_{100}), affecting the expression of cytochrome c and nanowires and affecting the R_V_. Acting as an electron shuttle early in incubation, AQDS can accelerate the reduction of iron oxide minerals by enhancing electron transport [38,39]. Later, the main function of AQDS was to complex with Fe(II) ions to avoid the excessive adsorption and accumulation of Fe(II) on the surface of iron oxide minerals and bacteria, hindering the reduction of Fe(III) but improving the Rv_._

Our findings reveal the role of different exposed facets of iron oxides in the process of vivianite formation by DIRB.

## 4. Conclusions

In this study, different exposure facets of iron oxide minerals as electron acceptors in the dissimilatory iron reduction-driven biomineralization process were proven to have different effects. In a phosphoric acid buffered system, Hem_{001} showed a higher reduction rate and degree of iron than Hem_{100}. *G. sulfurreducens* more easily dissolves Hem_{001} and Goe_H{110} with the same crystal structure, producing greater amounts of Fe(II) to combine with PO_4_^3−^, thus promoting the formation of more phosphorus crystal products. The addition of AQDS as an electron shuttle also had a great impact. The phosphorus crystallization products formed by the dissimilated iron reduction were mainly vivianite, and the size of vivianite crystals was affected by the different crystal surfaces of the iron oxides. The size of vivianite formed in the goethite system was larger than that of hematite. The size of vivianite formed in the Hem_{001} system was larger than that of Hem_{100}. The vivianite formed in the Goe_H{110} system had a larger aspect ratio than Goe_L{110}. The combined effect of the difference in expression of cytochrome c and nanowire-related genes, the difference in density of -OH sites, and the difference in solubility caused by different crystal surfaces of the ferric oxide is the potential mechanism by which *G. sulfurreducens* forms face-dependent vivianite. This work is of great significance for the sustainable ecological development of soil heavy metal solidification, organic matter dechlorination, and phosphorus fixation in sediments and soil systems.

## Data Availability

Datasets are available through the corresponding author upon reasonable request.

## Supplementary Materials

The following supporting information can be downloaded at: https://www.mdpi.com/article/10.3390/ijerph20054247/s1. References [9,11,16,40,41,42,43,44] are cited in the Supplementary Materials.
