# Supplementary material for: Facet Dependence of Biosynthesis of Vivianite from Iron Oxides by Geobacter sulfurreducens
Source: Int J Environ Res Public Health. 2023 Feb 27;20(5):4247. doi: 10.3390/ijerph20054247 (PMC10002410; doi:10.3390/ijerph20054247)
Supplement: Supplementary file 1 [file ijerph-20-04247-s001.zip › ijerph-2225628-supplementary.pdf]

Supporting Information

Facet-dependence of biosynthesis of vivianite from iron oxides by Geobacter sulfurreducens

Xiaoshan Luo [1,†], Liumei Wen [1,†], Lihua Zhou [2,*] and Yong Yuan [1]

1 Guangzhou Key Laboratory Environmental Catalysis and Pollution Control, Guangdong Key Laboratory of Environmental Catalysis and Health Risk Control, School of Environmental Science and Engineering, Institute of Environmental Health and Pollution Control, Guangdong University of Technology, Guangzhou 510006, China

2 Department of Pharmaceutical Engineering, School of Biomedical and Pharmaceutical Sciences, Guangdong University of Technology, Guangzhou 510006, China

* Correspondence: qhzhoulh@gdut.edu.cn

† Co-first authors.

Text S1. Synthesis of Iron Oxide Nanocrystals

The hematite nanoparticles (denoted as Hem_{001} and Hem_{100}) were synthesized according to previously reported methods [1-3]. In a synthesis of Hem_{001}, 1.64 g of $FeCl_3 \cdot 6H_2O$ was dissolved in 60.0 mL ethanol under vigorous magnetic stirring with a trace addition of ultrapure water (4.2 mL). 4.80 g of sodium acetate was added while stirring until completely dissolved. The mixture was sealed in a Teflon-lined stainless-steel autoclave (100 mL) and maintained at 180 °C for 12 h for solvothermal crystallization. The precipitates were centrifuged and washed with absolute ethanol and ultrapure water successively several times, and finally dried overnight at 60 °C. In a synthesis of Hem_{100}, 4.05 g of $FeCl_3 \cdot 6H_2O$ was added to 30 mL ultrapure water to form a 0.5 mol L^{-1} ferric chloride solution and then stirred for 10 min. After stirring, 1, 2-diaminopropane was added at a 1:1 volume ratio, and stirring was continued for 20 min. The solution was then transferred to a Teflon-lined stainless-steel autoclave and heated to 180 °C for 12 h for solvothermal crystallization. The precipitates were centrifuged and washed with absolute ethanol and ultrapure water successively several times, and finally dried by freeze-drying in a lyophilizer overnight. In order to remove any possible residual of organic solvents on the surface of the hematite, the hematite powders were calcined at 180 °C for 5 hours in air.

The goethite nanoparticles (denoted as Goe_H{110} and Goe_L{110}) were synthesized according to previously reported methods [4-6]. In a synthesis of Goe_H{110}, 250 mL of a 0.5 mol L^{-1} $FeCl_3 \cdot 6H_2O$ solution was neutralized with 200 mL of a 2.5 mol L^{-1} NaOH solution. The resulting suspension was aged in an oven at 70 °C for 60 h. The precipitates were washed to neutral pH using ultrapure water and finally dried at 50 °C. In a synthesis of Goe_L{110}, 22 mL of 10 mol L^{-1} NaOH solution was dropwise added to 200 mL of 40 mmol L^{-1} $FeSO_4 \cdot 7H_2O$ solution with magnetic stirring under an argon atmosphere. The mixture was then stirred continuously for 12 h while compressed air was passed into the solution. After reaction, the resultant solid was collected via centrifugation and washed with ultrapure water and absolute ethanol several times, and finally dried in an oven at 50 °C.

Text S2.

The phosphorus removal efficiency (R_P) and vivianite formation efficiency (R_V) was calculated as follows [7]:

$$R_{\mathrm{P}} = \frac{C_{\mathrm{SP}}}{C_0} \times 100\% \qquad \text{(S1)}$$

C_{SP} is the final solid P content, and C_0 is the initial PO_4^{3-} concentration.

$$R_{\mathrm{V}} = \frac{C_{\mathrm{V}}}{C_0} \times 100\% \qquad \text{(S2)}$$

C_V is the P content in vivianite and C_0 is the initial PO_4^{3-} concentration.

The crystallinity was calculated from the obtained diffraction patters by the equation below [8]:

$$\text{Crystallinity (\%)} = \frac{\text{the sum of the dominant peaks area}}{\text{total area}} \times 100\% \qquad \text{(S3)}$$

The specific surface area is calculated by the following formula:

$$\frac{\mathrm{P}}{\mathrm{v}(\mathrm{P_0}-\mathrm{P})} = \frac{1}{\mathrm{v_m C}} + \frac{\mathrm{C}-1}{\mathrm{v_m C}} \cdot \frac{\mathrm{P}}{\mathrm{P_0}} \qquad \text{(S4)}$$

Where P represents the partial pressure of nitrogen, P0 is the saturated vapor pressure of nitrogen at the liquid nitrogen temperature, V is the actual adsorption capacity of the sample, Vm is the saturated adsorption capacity of nitrogen monolayer, and C is the constant related to the adsorption capacity of the sample.

Table S1 Key physicochemical properties of the synthesized nanoparticles

Material	Particle size (nm)			Exposed facets	SA_{BET} (m^2 g^{-1})
	Length	Width	Thickness		
Hem_{100}	750	-	120	94.0% {100}	8.04
Hem_{001}	-	180	20	85.5% {001}	24.59
Goe_H{110}	1300	215	-	98.5% {110}<br>1.5% {021}	66.88
Goe_L{110}	120	35	-	82.3% {110}<br>17.7% {021}	76.01

Table S2 Product size, Fe/P and merit (FOM) results of the final product of each experimental group.

Groups	Mineral	product size (μm)		Fe/P	Fom
		Length	Width		
Hem_{100}	vivianite	1.5	0.6	1.52	28.8
Hem_{001}	vivianite	7.1	4.3	1.45	25.3
Goe_H{110}	vivianite	12.3	5.1	1.47	36.6
Goe_L{110}	vivianite	10.4	6.5	1.42	22.6

Figure S1

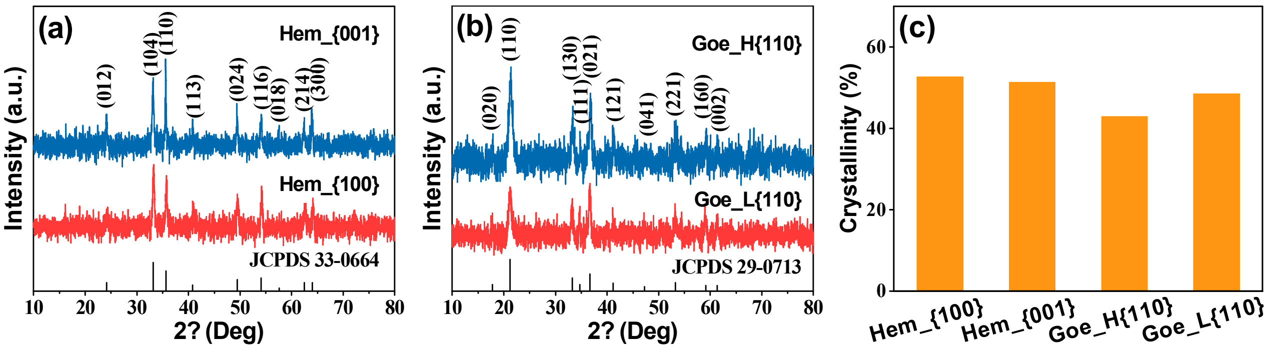

Figure S1 XRD patterns (a and b) and crystallinity (c) of iron (hydr) oxide nanoparticles. The crystallinity was calculated from the obtained diffraction patters by the equation in attached Text S2.

Figure S2

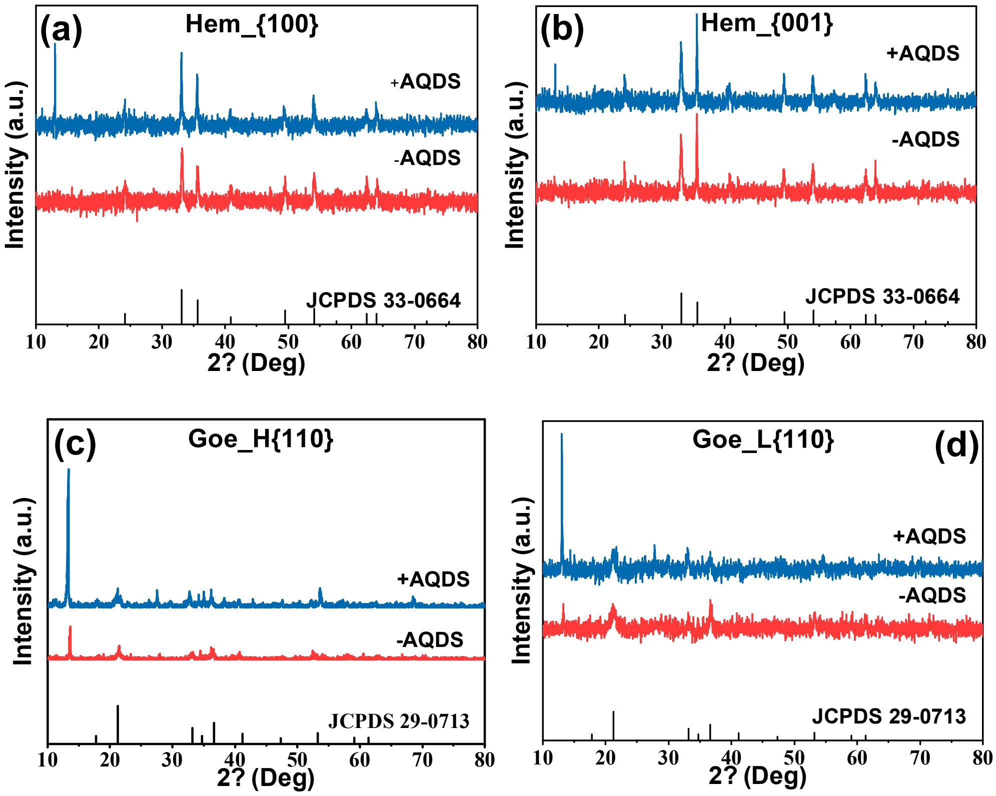

Figure S2 X-ray diffraction (XRD) patterns of iron (hydr) oxide nanoparticles after inoculation for 30 days with or without AQDS. XRD of (a) Hem_{100}, (b) Hem_{001}, (c) Goe_H{110} and (d) Goe_L{110}. Experimental conditions: bicarbonate−buffered (20mM); *Geobacter sulfurreducens* ($OD_{600} \approx 0.28$); 0 or 50 μM AQDS; 0.10 g/L iron (hydr)oxide nanocrystals.

Figure S3

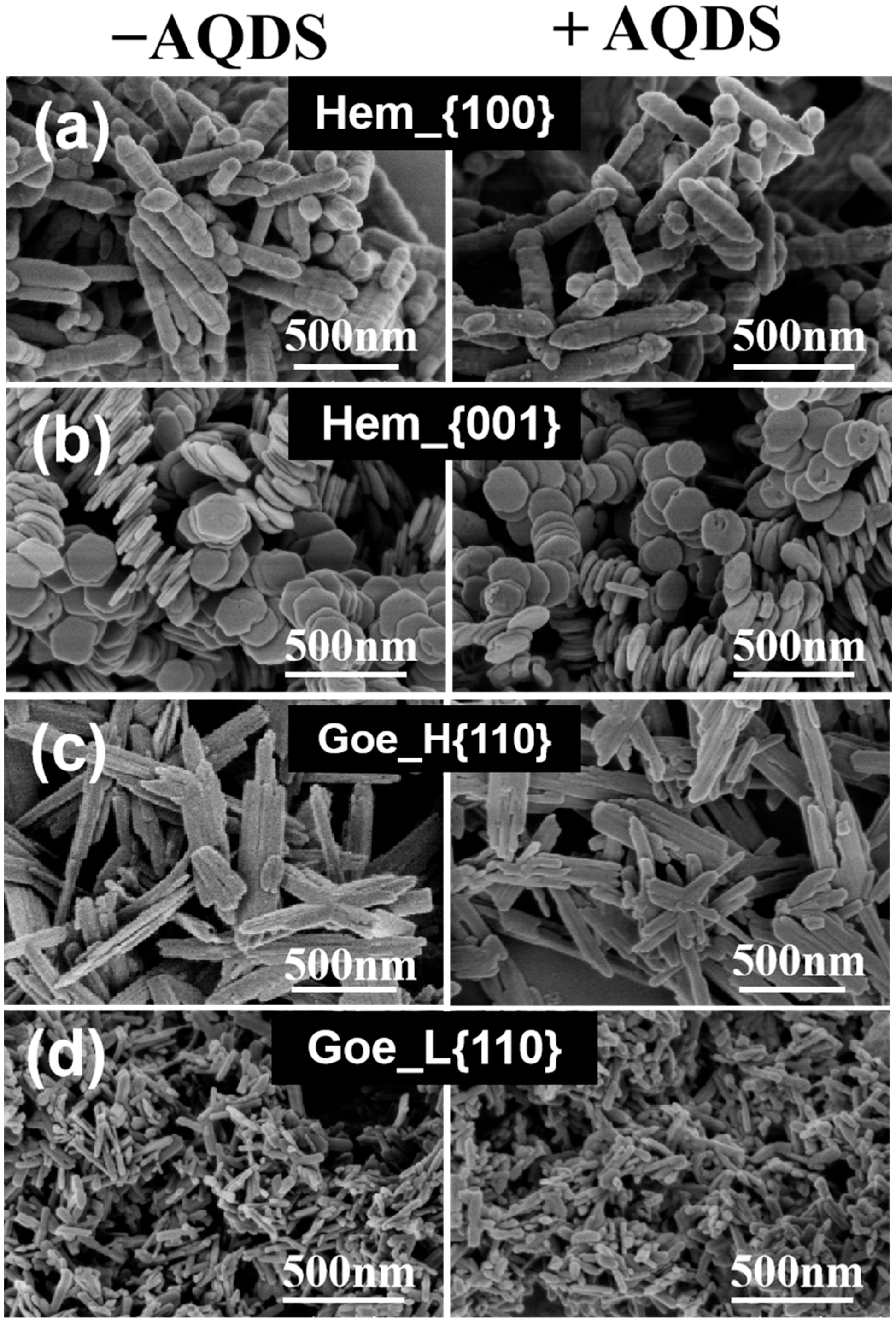

Figure S3 Scanning electron microscopy (SEM) of iron (hydr) oxide nanoparticles in separate setups, (before inoculation and 30 days after inoculation, with or without AQDS). (a) Hem_{100}; (b) Hem_{001}; (c) Goe_H{110} and (d) Goe_L{110}. Experimental conditions: bicarbonate−buffered (20mM); *Geobacter sulfurreducens* (OD_{600} ≈ 0.28); 0 or 50 μM AQDS; 0.10 g/L iron (hydr)oxide nanocrystals.

Figure S4

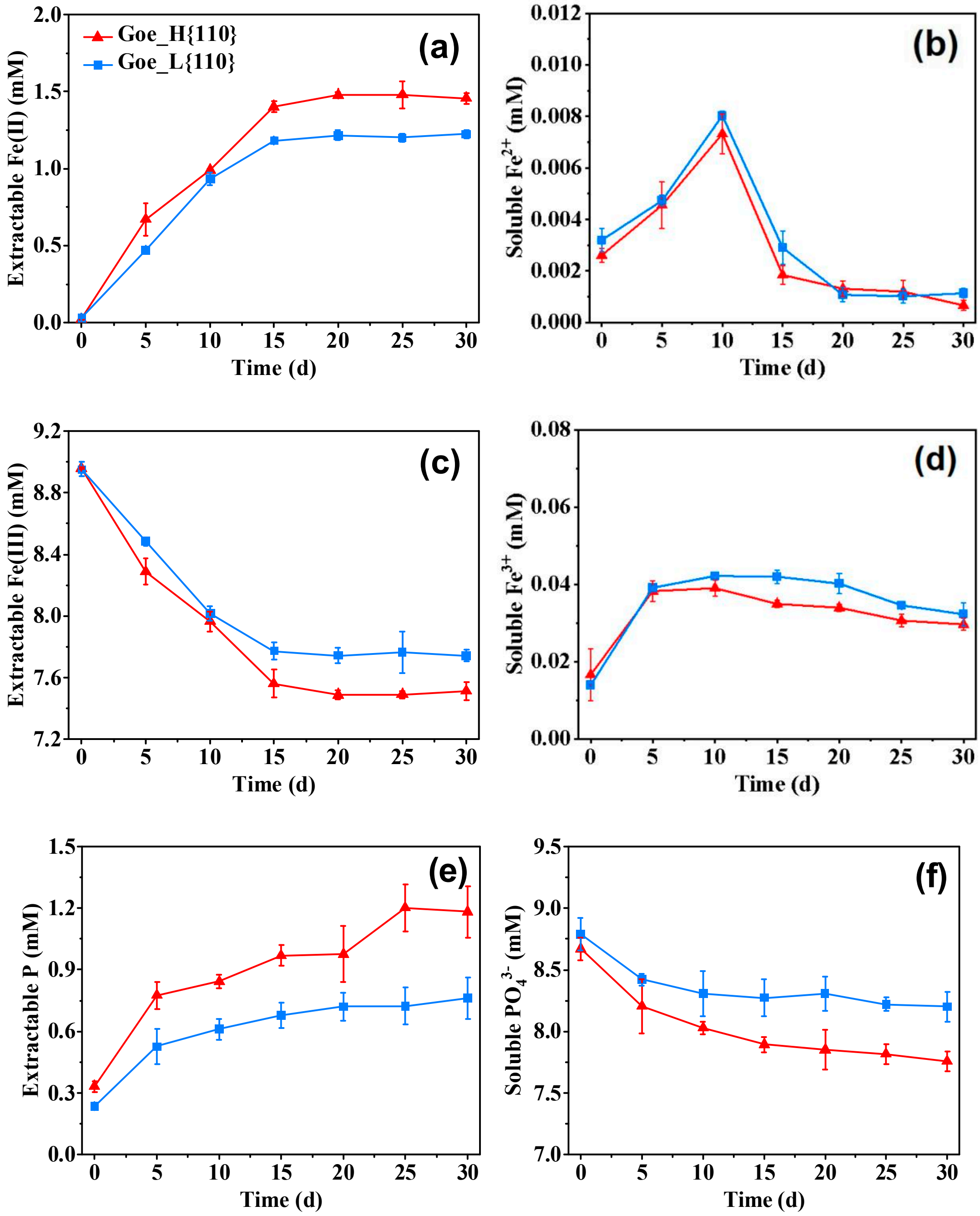

Figure S4 Variation of the (A) soluble Fe^{2+} content, (B) solid Fe(II) content, (C) soluble Fe^{3+} content, (D) solid Fe(III) content, (E) $PO_4{}^{3+}$ content, and (F) solid P content in goethite batches. Data corresponds to an average of biological triplicates, and the error bars indicate standard deviations. Experimental conditions: hydrophosphate−buffered (9mM); Geobacter sulfurreducens ($OD_{600} \approx 0.30$); iron (hydr)oxide nanocrystals (9mM).

Figure S5

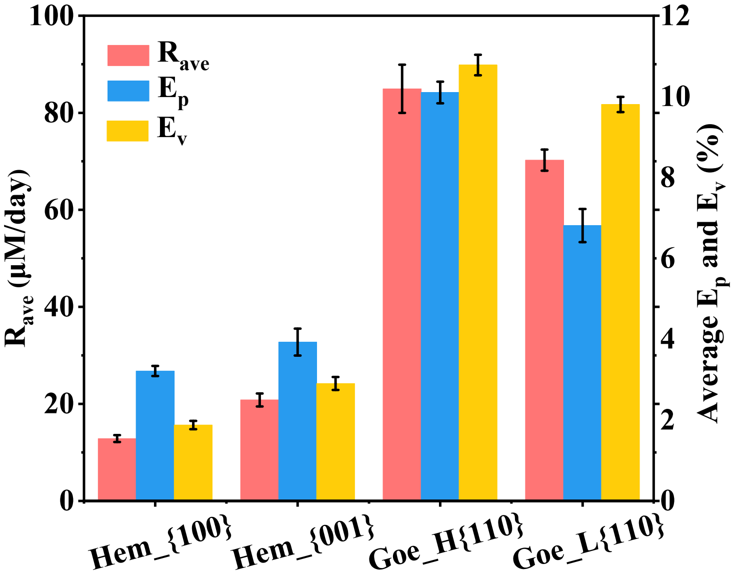

Figure S5 Dissimilatory iron reduction rate (R) of hematite and goethite systems, and the (c) average dissimilatory iron reduction rate (Rave), average phosphorus mineralization efficiency (Ep) and vivianite formation efficiency (Ev) in hematite and goethite systems. Data corresponds to an average of biological triplicates, and the error bars indicate standard deviations. Experimental conditions: hydrophosphate−buffered (9mM); Geobacter sulfurreducens ($OD_{600} \approx 0.30$); iron oxide nanocrystals (9mM).

Figure S6

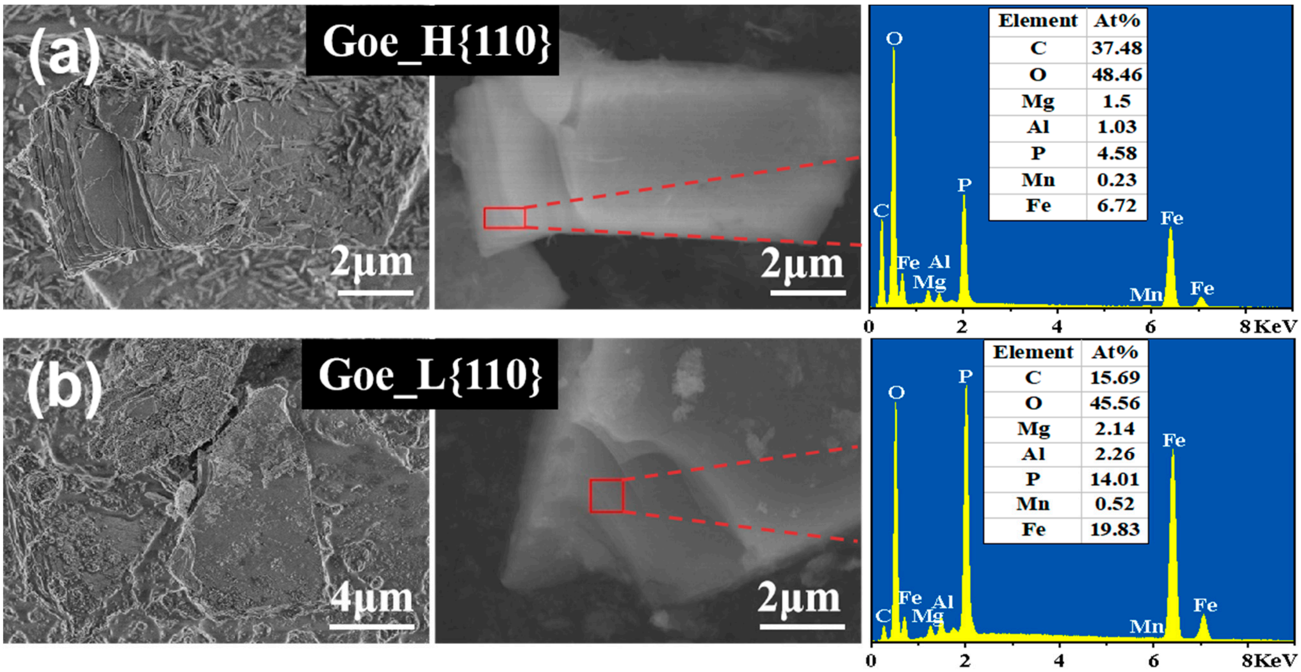

Figure S6 SEM image of the vivianite in (a) Goe_H{110}and (b) Goe_L{110} systems, and the corresponding EDS patterns.

Figure S7

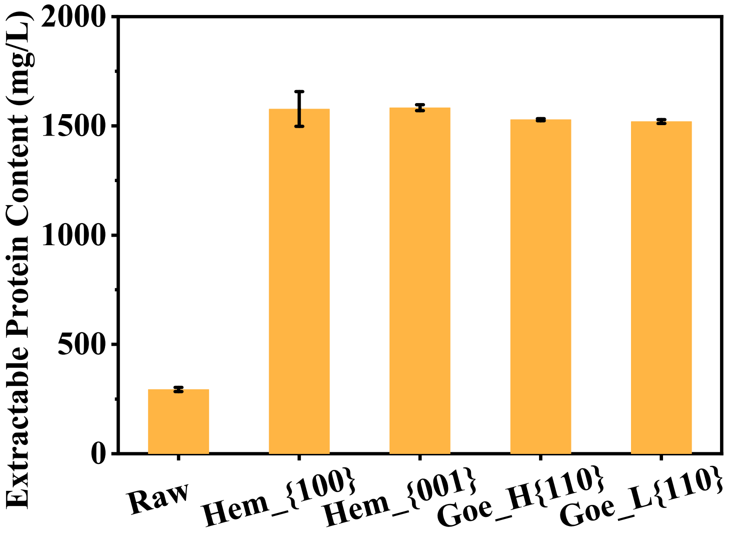

Figure S7 Protein contents of initial and 30 days after inoculation in various sample sets. Experimental conditions: hydrophosphate−buffered (9mM); Geobacter sulfurreducens ($OD_{600} \approx 0.30$); iron (hydr)oxide nanocrystals (9mM).

1 Lv J.; Miao Y.; Huang Z.; Han R.; Zhang S. Facet-mediated adsorption and molecular fractionation of humic substances on hematite surfaces. Environ. Sci. Technol. **2018**, *52(20)*, 11660-11669.

2 Chen L.; Yang X.; Chen J.; Liu J.; Wu H.; Zhan H.; Liang C.; Wu M. Continuous shape and spectroscopy tuning of hematite nanocrystals. Inorg Chem. **2010**, *49(18)*, 8411-8420.

3 Li Z.; Lai X.; Wang H.; Mao D.; Xing C.; Wang D. Direct hydrothermal synthesis of single-crystalline hematite nanorods assisted by 1,2-propanediamine. Nanotechnology. **2009**, *20(24)*, 245603.

4 Li T.; Zhong W.; Jing C.; Li X.; Zhang T.; Jiang C.; Chen W. Enhanced hydrolysis of p-nitrophenyl phosphate by iron (hydr)oxide nanoparticles: Roles of exposed facets. Environ. Sci. Technol. **2020**, *54(14)*, 8658-8667.

5 Lv J.; Zhang S.; Wang S.; Luo L.; Cao D.; Christie P. Molecular-Scale Investigation with ESI-FT-ICR-MS on Fractionation of Dissolved Organic Matter Induced by Adsorption on Iron Oxyhydroxides. E nviron. Sci. Technol. **2016**, *50(5)*, 2328-2336.

6 Kakuta S.; Numata T.; Okayama T. Shape effects of goethite particles on their photocatalytic activity in the decomposition of acetaldehyde. Catal Sci Technol. **2014**, *4(1)*, 164-169.

7 Wang, S.; Wu, Y.; An, J.; Liang, D.; Tian, L.; Zhou, L.; Wang, X.; Li, N. Geobacter autogenically secretes fulvic acid to facilitate the dissimilated iron reduction and vivianite recovery. Environ. Sci. Technol. **2020***, 54,* 10850-10858.

8 Akın, S.Ş.; Magalhaes, D.; Kazanc, F. A study on the effects of various combustion parameters on the mineral composition of Tunçbilek fly ash. Fuel. **2020**, *275*, 117881.
